# Targeted therapy for cisplatin‐resistant lung cancer via aptamer‐guided nano‐zinc carriers containing USP14 siRNA

**DOI:** 10.1002/mco2.237

**Published:** 2023-04-06

**Authors:** Xinmin Zhao, Xianghua Wu, Huijie Wang, Songtao Lai, Jialei Wang

**Affiliations:** ^1^ Department of Thoracic Medical Oncology Fudan University Shanghai Cancer Center Shanghai China; ^2^ Department of Oncology Shanghai Medical College, Fudan University Shanghai China; ^3^ Department of Radiation Oncology Fudan University Shanghai Cancer Center Shanghai China; ^4^ Shanghai Key Laboratory of Radiation Oncology Shanghai China

**Keywords:** cisplatin‐resistance, nano‐zinc carriers, non‐small cell lung cancer, small interfering RNA, targeted therapy

## Abstract

Cisplatin (DDP) is a common therapeutic option for non‐small cell lung carcinoma (NSCLC). However, some patients fail to respond to the DDP chemotherapy. Therefore, identifying novel biomarkers to improve the diagnosis and treatment of NSCLC is important. Ubiquitin‐specific protease (USP14) is involved in various pathological conditions including cancer; however, the role of USP14 in NSCLC remains elusive. The SELEX technology was used to identify aptamers that specifically recognize DDP‐resistant lung cancer cells and couple them with nano‐zinc (zinc hydroxide, Zn(OH)_2_) carriers. USP14 levels were higher in DDP‐resistant lung cancer compared to DDP‐sensitive lung cancer. The survival rate of lung cancer patients with increased USP14 expression was significantly lower than the survival rate of patients with low USP14 expression. Silencing USP14 increased the tumor antagonistic action of DDP in A549 cisplatin‐resistant (A549/DDP) cells, while USP14 overexpression decreased the antagonist effects. Aptamer‐targeted nano‐zinc carriers were loaded with USP14 siRNA to target DDP‐resistant lung cancer cells. Aptamer‐targeted nano‐zinc carriers containing USP14 siRNA increased the antitumor effects of DDP in A549/DDP cells and mice bearing A549/DDP cells. These results indicate that aptamer‐guided nano‐zinc carriers may be a potent carrier for the precise treatment of drug‐resistant tumors.

## Graphical abstract

The ligands are combined with nano‐zinc hydroxide carriers to guide aptamer and siUSP14 to specifically bind to the surface of DDP‐resistant A549/DDP cells. After internalization of the paired aptamer and carriers, intracellular pH changes cause the active release of the siUSP14, followed by inactivation of epidermal growth factor receptor (EGFR)/PI3K/protein kinase B (AKT) signaling pathway, resulting in decreased DDP resistance in A549/DDP cells.

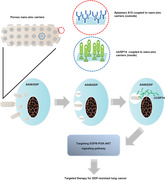

## 1 INTRODUCTION

Lung cancer has a high worldwide prevalence and is usually malignant, with the highest cancer‐related mortality.^1^, ^2^ Lung cancers can be grossly classified into non‐small cell lung carcinoma (NSCLC), accounting for about 85% of lung cancers, and small cell lung carcinoma, which is less prevalent. NSCLC can be further divided into adenocarcinoma, squamous cell carcinoma, and large cell carcinoma.^3^, ^4^ Lung cancer remains asymptomatic in the initial stages; hence, a majority of patients have already reached the tumor progression stage when diagnosed.^5^ Classical multi‐course chemotherapy and chemotherapy combined with radiotherapy prolong overall survival in advanced lung cancer. However, the side effects of the chemotherapy due to cytotoxicity in normal cells can be unbearable and the tumor cells may develop resistance to chemotherapy drugs. Thus, the application of these therapies is problematic.^6^ Therefore, the exploration of effective adjuvant therapies is crucial in improving patient prognosis.

Cisplatin (DDP) has been a significant tumor chemotherapy against various solid malignant tumors, including lung adenocarcinoma, since it was approved by the Food and Drug Association in 1978.^7^ Platinum chemotherapy drugs, represented by DDP, are antitumor agents. DDP has gradually become the standard chemotherapy method for chronic lung adenocarcinoma and postoperative grade II and III lung adenocarcinoma.^8^ Although lung adenocarcinoma reacts well in the initial stage of standard cisplatin chemotherapy, significant DDP resistance develops before chemotherapy (primary resistance) or during the course of chemotherapy (acquired resistance),^9^ resulting in treatment failure. Rapid DDP resistance is the main obstacle to recovery during long‐term follow‐up of lung adenocarcinoma patients,^10^, ^11^ with an overall five‐year survival of just 16%.^12^ Therefore, improving the efficacy of DDP chemotherapy and sequential treatment after the failure of the DDP chemotherapy is crucial to saving patient lives.

Deubiquitination (DUB) plays an important regulatory function in cell survival.^13^, ^14^, ^15^ Ubiquitin‐specific protease 14 (USP14), belonging to the USP protein family, reacts with the 26S proteasome complex and enhances DUB in the proteasome 19S regulatory pathway. USP14 influences the incidence of breast cancer, lung adenocarcinoma, multiple myeloma, and other types of tumors.^16^ Overexpression of USP14 in lung adenocarcinoma promotes proliferation via the accumulation of β‐catenin^17^ and regulates lung tumorigenesis through cell apoptosis and autophagy pathways.^16^ USP14 is critical for NSCLC migration via deubiquitylation and stabilization of Acf7.^18^ Suppression of USP14 induces NSCLC sensitivity to gefitinib.^19^ However, the role of USP14 in lung cancer sensitivity to DDP is unclear.

With the rapid development of nanotechnology, nanoforms of metal oxides are widely used in disease diagnosis, drug delivery, and biological imaging. Among the metal (Au, Ag, etc.) or metal oxide (TiO_2_, CuO, FeO) nanoparticles, zinc oxide nano‐zinc carriers are low‐cost, easily available, multifunctional inorganic nanomaterials that can be produced on a large scale. In addition, zinc oxide nano‐zinc carriers exhibit low‐skin permeability, a wide UV reflection range, and wide antibacterial activity.^20^ Zinc oxide nanocarriers may be selective killers of highly proliferative cells; thus, they have great potential in clinical anticancer therapy.^21^ The biocompatibility of zinc oxide nanocarriers can be improved by modification and doping methods, and zinc oxide nanocarriers can target cancer cells. Precise targeting of zinc oxide nanocarriers to cancer cells can be achieved by loading them with specific ligands of receptors that are highly expressed in cancer cells.^22^

No matter how DDP‐resistant NSCLC is treated, accurately targeting cancer cells is a key problem. Aptamers (AMs) are single‐stranded deoxyribonucleic acids or ribonucleic acids that can potentially bind to targets, including cells, tissues, proteins, peptides, small molecules, or bacterial organisms, with high specificity and affinity using Systematic Evolution of Ligands by Exponential Enrichment (SELEX) in vitro.^23^, ^24^ Compared with antibodies, AMs are cheaper, easier to use, and more stable.^25^ In addition, AMs possess excellent tissue permeability and can easily enter cells, even in the absence of immunogenicity. Recently, AMs targeted to proteins have attracted wide attention in the fields of molecular biology, biotechnology, and biomedicine.^26^, ^27^, ^28^ Haghighi et al. developed AMs that bind the human B‐cell surface protein CD20, which may overtake antibodies in the diagnosis and treatment of immune deficiency, autoimmune diseases, leukemia, and lymphoma.^29^ He et al. developed two AMs that selectively bind to two glycoproteins on the surface of a paclitaxel‐resistant ovarian carcinoma cell line with high selectivity, strong affinity, and good nuclease tolerance in serum. These AMs can be utilized in the diagnosis of drug‐resistant ovarian carcinoma.^30^

In this study, we identified AMs specifically targeted to DDP‐resistant NSCLC cells, and, subsequently, coupled the AMs with nano‐zinc carriers. The nano‐zinc carriers were loaded with USP14 siRNA. When tumor cells endocytose the nano‐zinc carriers, high concentrations of USP14 siRNA are released in cells to effectively treat NSCLC.

## 2 RESULTS

### 2.1 USP14 expression increased in NSCLC patients with DDP resistance

**FIGURE 1:**
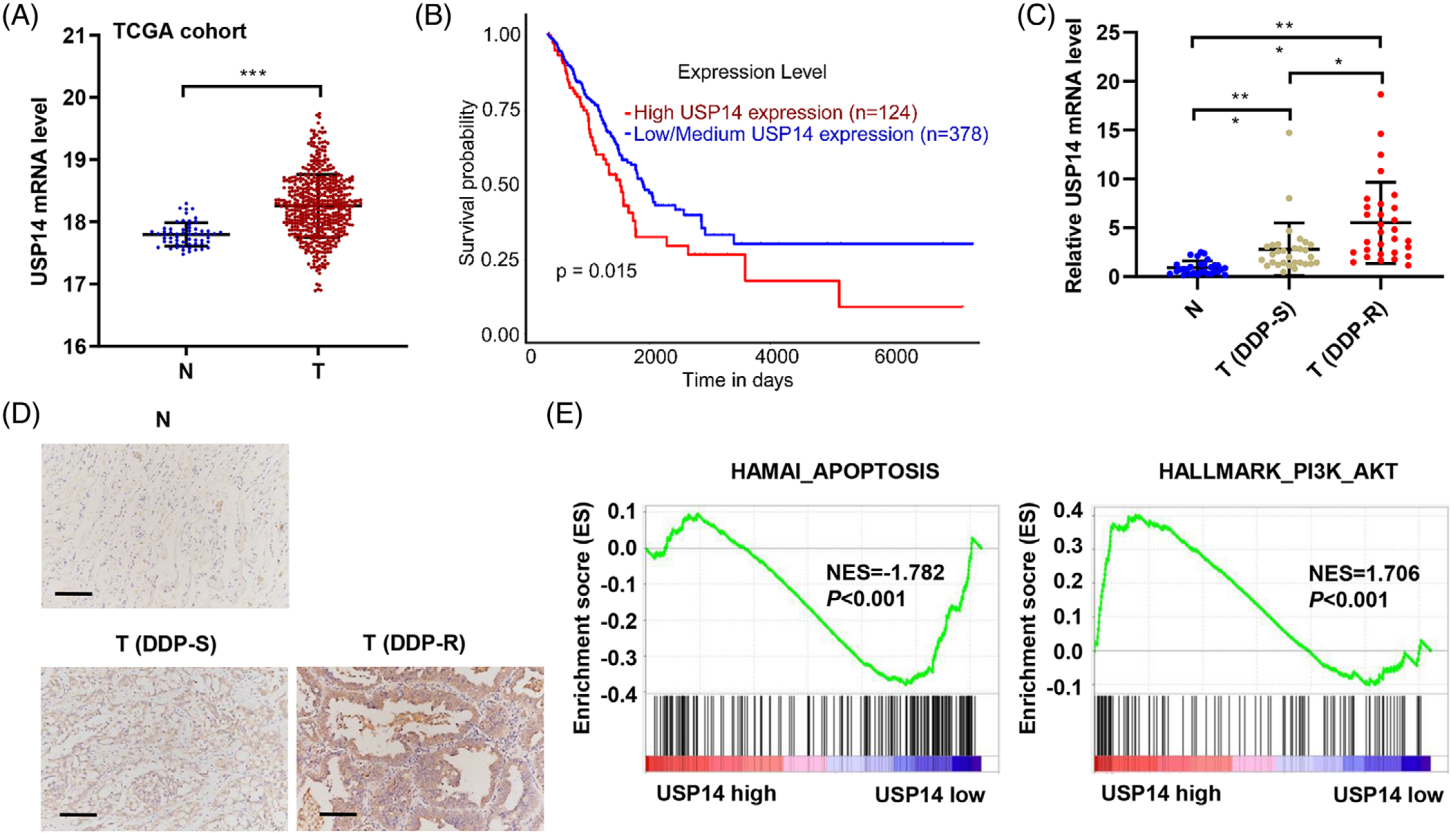
USP14 expression is increased in lung cancer tissues and correlates with the responsiveness of lung adenocarcinoma tissue to DDP therapy. (A) USP14 expression in lung cancer tissues (T; *n* = 526) and normal lung tissues (N, *n* = 59) from TCGA dataset. (B) The overall survival rate of patients with lung cancer from TCGA dataset. The expression of USP14 in lung cancer DDP‐sensitive (*n* = 30) and DDP‐resistant (*n* = 30) tissues and normal lung (*n* = 30) tissues from a hospital cohort was quantified by (C) qRT‐PCR and (D) IHC. Scale bar, 100 μm. (E) GSEA demonstrated that USP14 expression was associated with apoptosis and the AKT signaling pathway. Data are presented as means ± SD. *P < 0.05, ***P < 0.001. DDP, Cisplatin; GSEA, gene set enrichment analysis; IHC, immunohistochemistry; qRT‐PCR, quantitative real‐time polymerase chain reaction; TCGA, The Cancer Genome Atlas; USP, ubiquitin‐specific protease.

To analyze the clinical relevance of USP14, the mRNA levels of USP14 in human lung cancer tissue and adjacent normal tissue were measured. The mRNA levels of USP14 were higher in lung cancer tissues compared with the levels in normal lung tissues in The Cancer Genome Atlas (TCGA) database (Figure 1A). The survival rate of people with high USP14 expression was lower than the survival rate of people with low USP14 expression in the TCGA database (Figure 1B). The expression of USP14 in tumor and normal lung tissues from subjects with lung cancer in a hospital cohort was measured by quantitative real‐time polymerase chain reaction (qRT‐PCR) and immunohistochemistry (IHC). As shown in Figures 1C,D, USP14 mRNA and protein levels were higher in the lung tissues of patients with lung cancer, especially in DDP‐resistant patients, compared with the levels in normal lung tissues. Gene set enrichment analysis (GSEA) showed that USP14 expression was related to apoptosis and the AKT signaling pathways (Figure 1E). These data suggest that USP14 is associated with DDP resistance in patients with lung cancer.

### 2.2 USP14 regulates cell viability, apoptosis, and DDP resistance

**FIGURE 2:**
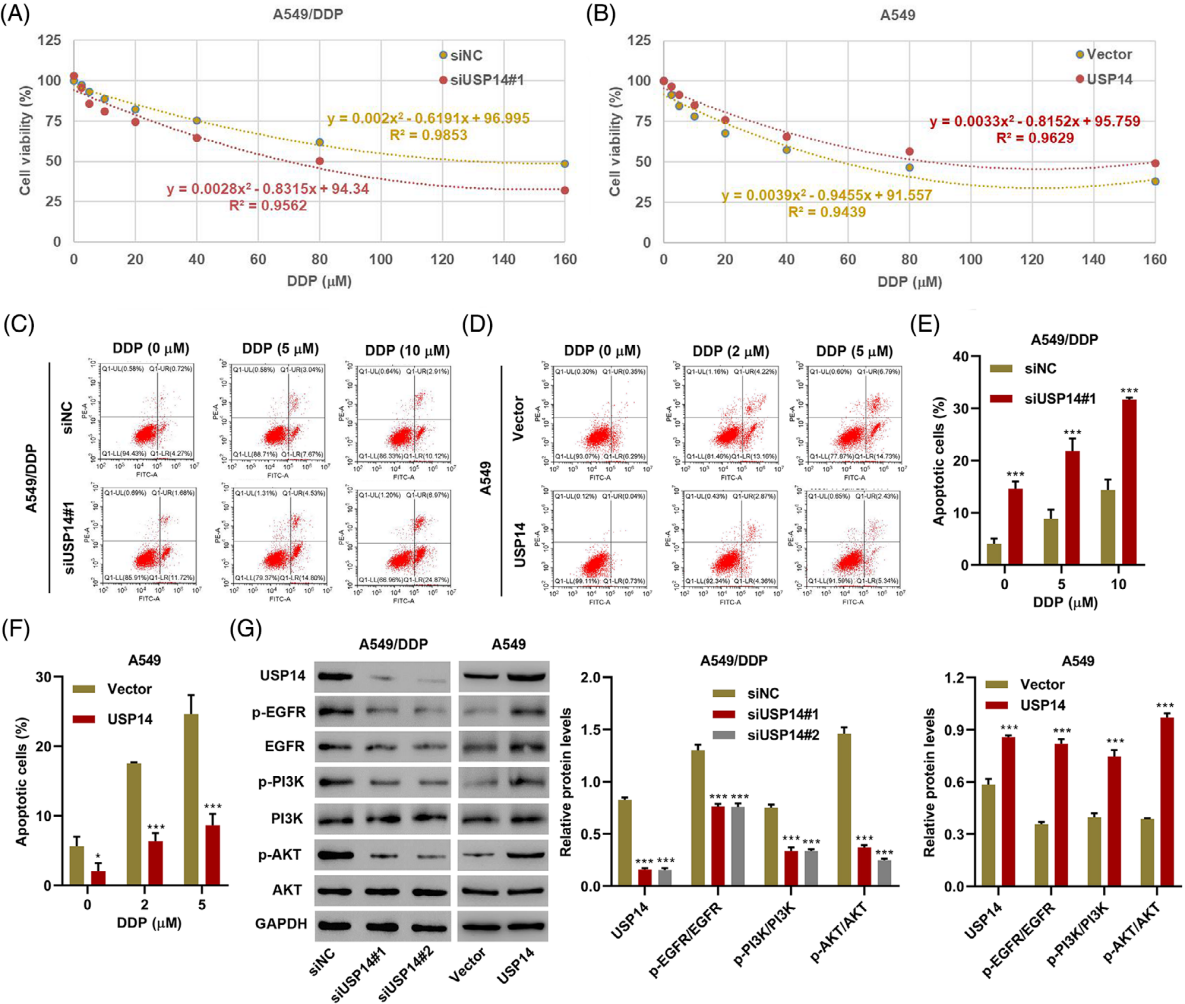
USP14 regulates cell viability, apoptosis, and DDP resistance in A549/DDP and A549 cells. (A, B) A549/DDP and A549 cell viability was measured 48 h after treatment with different concentrations of DDP. (C–F) Cell apoptosis and (G) expression of USP14, p‐EGFR, EGFR, p‐PI3K, PI3K, AKT, and p‐AKT was measured in A549/DDP cells transfected with USP14 siRNA (siUSP14#1 or siUSP14#2) and A549 cells transduced with USP14 expression plasmids followed by DDP treatment. Data are presented as means ± SD (*n* = 3). *P < 0.05, **P < 0.01, ***P < 0.001 compared with shNC or vector. DDP, Cisplatin; USP, ubiquitin‐specific protease.

To investigate the function of USP14 in lung cancer, two siRNAs targeting USP14 (siUSP14#1 and siUSP14#2) were transfected into A549/DDP cells and a USP14 expression vector was transduced into A549 cells. DDP cytotoxicity in A549/DDP and A549 cells was shown in Figure 2A and 2B, respectively. DDP with IC50 of 133.3 and 63.7 μM was obtained for A549/DDP cells with siNC or siUSP14#1 transfection, respectively (Figure 2A). DDP with IC50 of 57.7 and 86.2 μM was obtained for A549 cells with empty vector or USP14 expression vector transduction, respectively (Figure 2B). Moreover, apoptosis rates were higher in A549/DDP cells with relatively low USP14 expression compared with the rates in control cells (siNC) (Figure 2C,E). Apoptosis rates were lower in A549 cells with high USP14 expression compared with the rates in control cells (Vector) (Figure 2D,F). More importantly, low USP14 expression sensitized A549/DDP cells to DDP, while high USP14 expression desensitized A549 cells to DDP (Figure 2A–F). Western blots showed that the expression of USP14 positively correlated with the expression of p‐EGFR, EGFR, p‐PI3K, and p‐AKT (Figure 2G), suggesting that USP14 silencing inhibits DDP resistance via the EGFR/PI3K/AKT signaling pathway. Due to the similar effects of siUSP14#1 and siUSP14#2 on USP14 expression and EGFR/PI3K/AKT signaling pathway, siUSP14#1 was therefore selected randomly for subsequent experments.

### 2.3 USP14 interacts with EGFR and facilitates deubiquitination of EGFR

**FIGURE 3:**
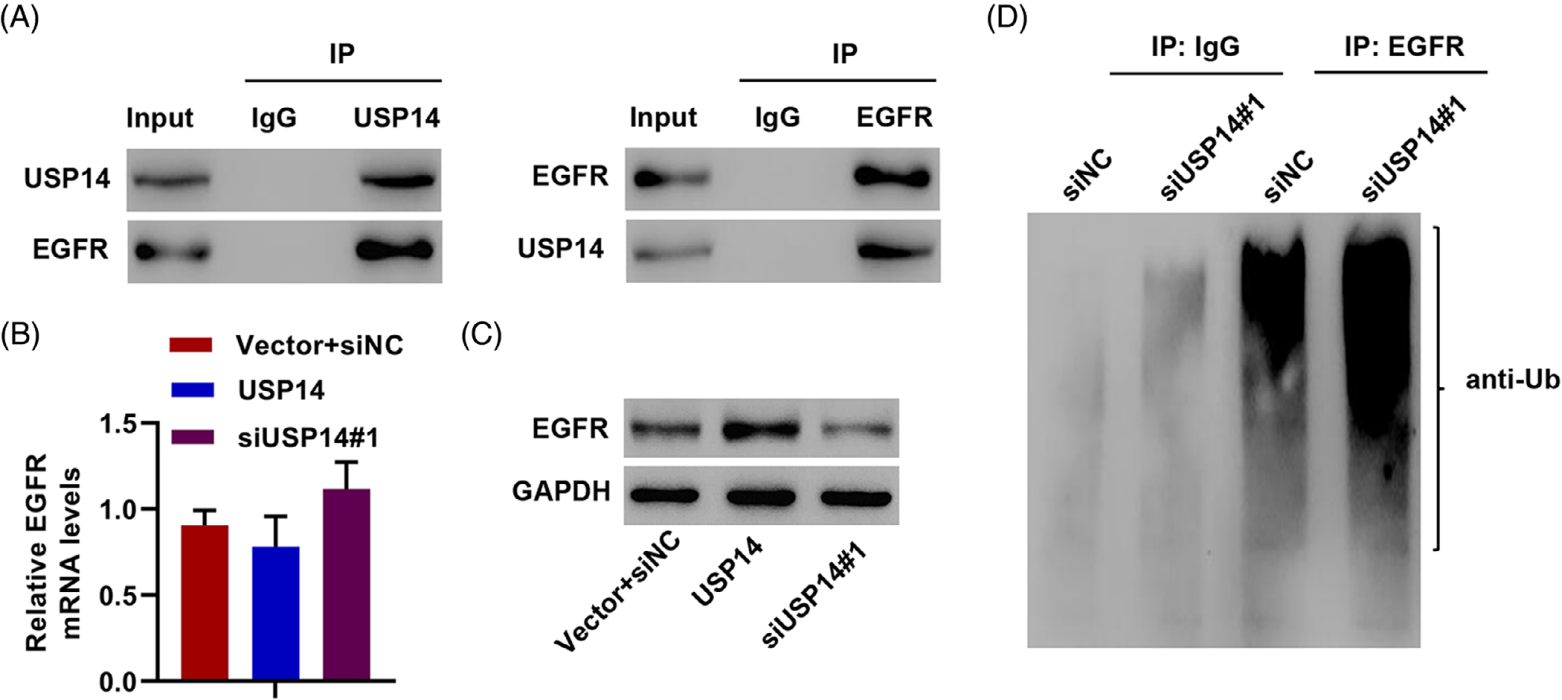
USP14 induces deubiquitination of EGFR. (A) Western blotting after immunoprecipitation with IgG control, anti‐USP14, or anti‐EGFR antibodies. EGFR (B) mRNA and (C) protein levels in A549/DDP cells transduced with USP14 expression plasmids or transfected with siUSP14#1. (D) A549/DDP cells transfected with siUSP14#1 were immunoprecipitated with EGFR or IgG antibodies and ubiquitination was assessed by the Western blot. Data are presented as means ± SD (*n* = 3). USP, ubiquitin‐specific protease.

USP14 is a DUB enzyme, and EGFR can be regulated by ubiquitination and DUB.^31^ Thus, we hypothesized that USP14 regulates EGFR protein levels via DUB. USP14‐EGFR interaction was confirmed using co‐immunoprecipitation (Figure 3A). Silencing USP14 significantly decreased EGFR protein levels, but not mRNA levels, and overexpressing USP14 remarkably increased EGFR protein expression but not EGFR mRNA in A549/DDP cells (Figure 3B,C). USP14 induced EGFR DUB to increase EGFR protein in A549/DDP cells (Figure 3D). These results demonstrate that USP14 interacts with EGFR and induces EGFR DUB.

### 2.4 Screening and characteristics of AM binding to A549/DDP using Cell‐SELEX

**FIGURE 4:**
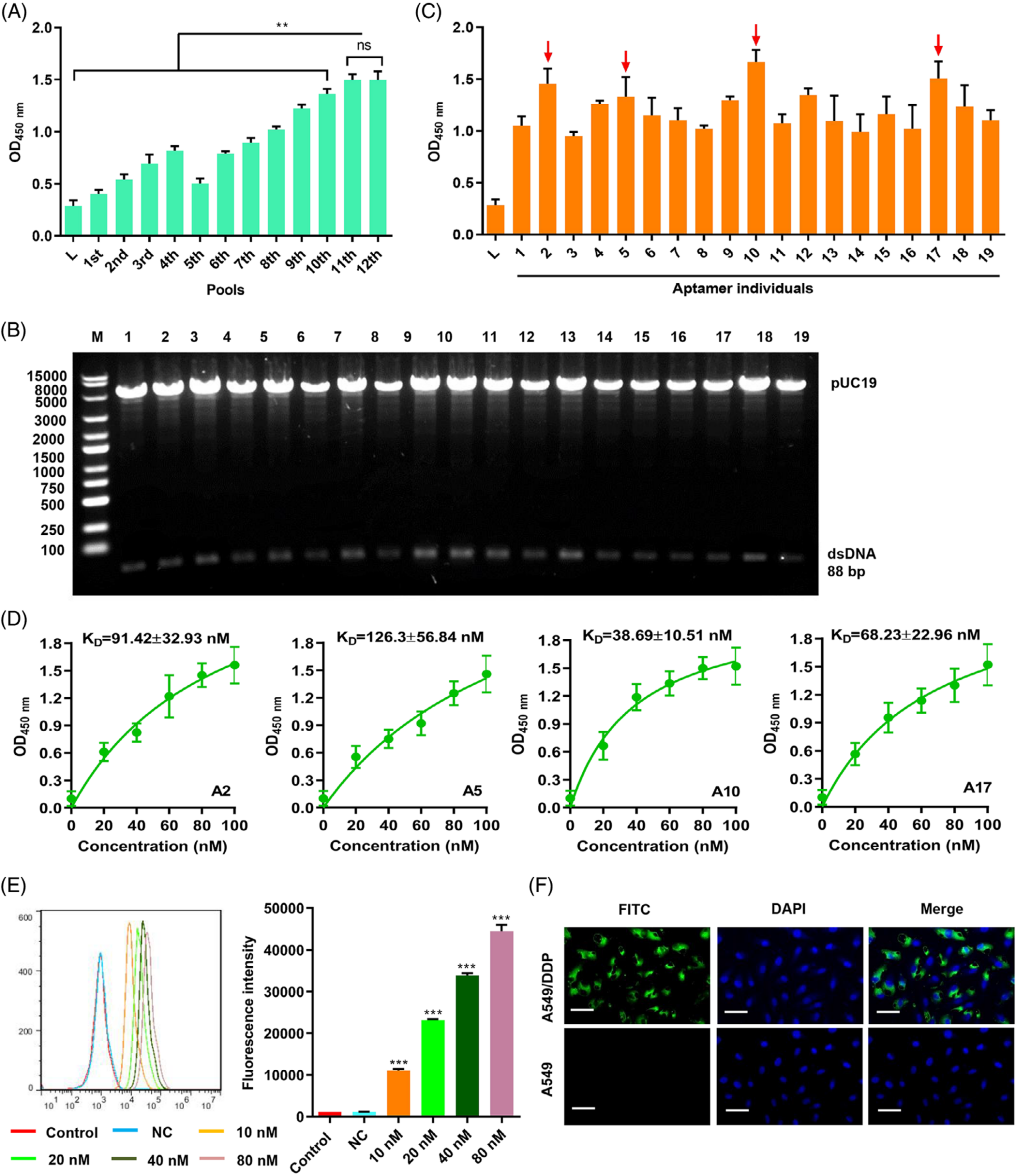
Cell‐SELEX for the identification of A549/DDP‐specific AM. (A) Binding capability of the enriched pools to A549/DDP cells determined by enzyme‐linked oligonucleotide assay. (B) Identification of dsDNA library amplified from the pool 11 library. (C) Binding of the AM candidates (A1–A19) to A549/DDP cells determined by enzyme‐linked oligonucleotide assay (red arrows indicated the AM candidates with higher binding forces). (D) Dissociation constant of aptamer candidates (A2, A5, A10, and A17) in A549/DDP cells was determined by enzyme‐linked oligonucleotide assay. (E) Binding of the different concentrations of FITC‐labeled aptamer 10 (A10) to A549/DDP cells detected by flow cytometry. (F) Subcellular localization of A10 in A549/DDP and A549 cells visualized using FITC‐labeled A10. Scale bar, 50 μm. Data are presented as means ± SD (*n* = 3). **P < 0.01, ***P < 0.001 in comparison to 11th, 12th, or NC. DDP, Cisplatin; SELEX, Systematic Evolution of Ligands by Exponential Enrichment.

Accurate targeting is the key to improving therapeutic effects. Therefore, we used Cell‐SELEX to screen for AMs in 12 pools (Figure S1). The binding forces of ssDNA from the 12 pools were compared using a cell‐enzyme‐linked oligonucleotide assay. The binding forces from the 11th and 12th pools were not significantly different (Figure 4A). The ssDNA from the 11th pool was amplified into dsDNA using PCR, and the dsDNA library was inserted into the pUC19 plasmid and transformed into *E. coli* DH5ɑ. Recombinant plasmids from colonies of *E. coli* DH5ɑ were identified by digestion with endonuclease enzyme (*Eco*R I and *Sac* I) (Figure 4B). The plasmids from positive colonies were amplified into ssDNA with unique sequences and binding force was compared using asymmetrical PCR. Four AMs (numbers 2, 5, 10, and 17) showed higher binding forces than the other AMs (Figure 4C). The *Kd* values of the four AMs were measured using cell‐ELONA and number 10 exhibited the highest binding force, with a *Kd* of 38.69 ± 10.51 nM (Figure 4D). The binding of fluorescein thiocyanate (FITC)‐labeled AM 10 (A10) to A549/DDP cells was dose‐dependently increased, as determined by flow cytometry (Figure 4E). The subcellular localization of A10 in A549/DDP and A549 cells was visualized using FITC‐labeled A10. Labeled A10 bound specifically to A549/DDP (Figure 4F), suggesting that A10 is a successful tool for targeting A549/DDP cells.

### 2.5 Aptamer‐siRNA nano‐zinc carriers regulate DDP resistance in A549/DDP cells

**FIGURE 5:**
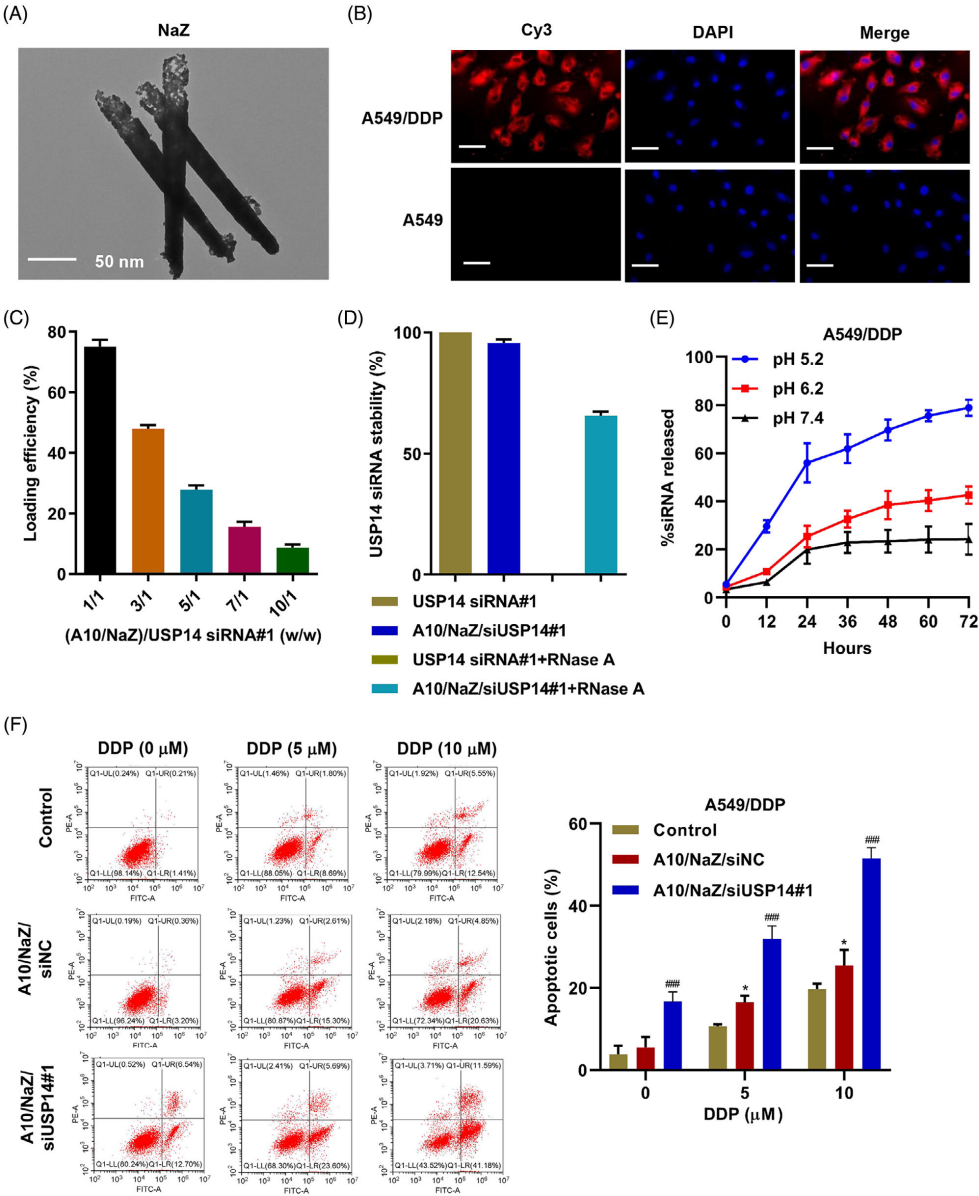
Aptamer‐siRNA nano‐zinc carriers regulate DDP resistance in A549/DDP cells. (A) Transmission electron microscopy of nanometer zinc carrier (NaZ). (B) The subcellular localization of A10 functionalized NaZ loaded with siUSP14#1 (A10/NaZ/siUSP14#1) in A549/DDP and A549 cells visualized using Cy3‐labeled siUSP14#1. Scale bar, 50 μm. (C) USP14 siRNA loading efficiency at various A10/NaZ to siUSP14#1 weight ratios. (D) Protection of siUSP14#1 from RNase A digestion was evaluated by incubating 5 pmol of siUSP14#1 alone or complexed with A10/NaZ with 5 mU RNase A at 37°C for 2 h. (E) Release kinetics of siUSP14#1 from A10/NaZ at different pHs with continuous agitation at 37°C. (F) Cell apoptosis of A549/DDP cells treated with DDP and A10/NaZ/siUSP14#1. Data are presented as mean ± SD (*n* = 3). *P < 0.05 compared to the control. ^###^ *P* < 0.001 compared with A10/NaZ/siNC. DDP, Cisplatin.

Nanotube zinc particles were designed as siUSP14 carriers in A10 oriented cells using the cell's phagocytic ability to internalize the nano‐zinc carriers and release siUSP14 inside the cell. The nano‐zinc carriers had a long (120–300 nm) cylindrical porous structure, as determined by the scanning electron microscopy (Figure 5A). To verify the targeting of this system, aptamer A10 was coupled to nano‐zinc carriers, and Cy3‐labeled siUSP14#1 was loaded into the nano‐zinc carriers. The modified nano‐zinc carriers were incubated with A549 and A549/DDP cell lines and the cells were observed using laser confocal microscopy. The aptamer A10 coupled with nano‐zinc carriers loaded with Cy3‐labeled siUSP14#1 bound specifically to A549/DDP cells (Figure 5B). Figure 5C shows the siUSP14#1 loading efficiency of the nano‐zinc carriers at various nano‐zinc carriers/siUSP14#1 weight ratios; maximum siUSP14 loading efficiency was observed at a weight ratio of 1/1. As shown in Figure 5D, the treatment of free siUSP14#1 with RNase A for 2 h resulted in the complete degradation of siRNA. However, nano‐zinc carriers increased the stability of siUSP14#1. In addition, the release profile of siUSP14#1 from nano‐zinc carriers was determined at pH values of 5.2, 6.2, and 7.4. We observed a 72 h sustained release of siUSP14 from the nano‐zinc carriers at different pH values (Figure 5E). At a relatively low pH of 5.2, 79% of siUSP14#1 was discharged from the nano‐zinc carriers in 72 h. In contrast, at a physiological pH of 7.4, only 24% of the siUSP14#1 was discharged in 72 h. When the aptamer A10‐coupled nano‐zinc carriers loaded with siUSP14#1 were internalized, DDP induced dose‐dependent apoptosis (Figure 5F). These results demonstrate that aptamer‐siRNA nano‐zinc carriers inhibit DDP resistance in A549/DDP cells.

### 2.6 Antitumor efficacy of aptamer‐siRNA nano‐zinc carriers in a lung cancer mouse model bearing A549/DDP cells

**FIGURE 6:**
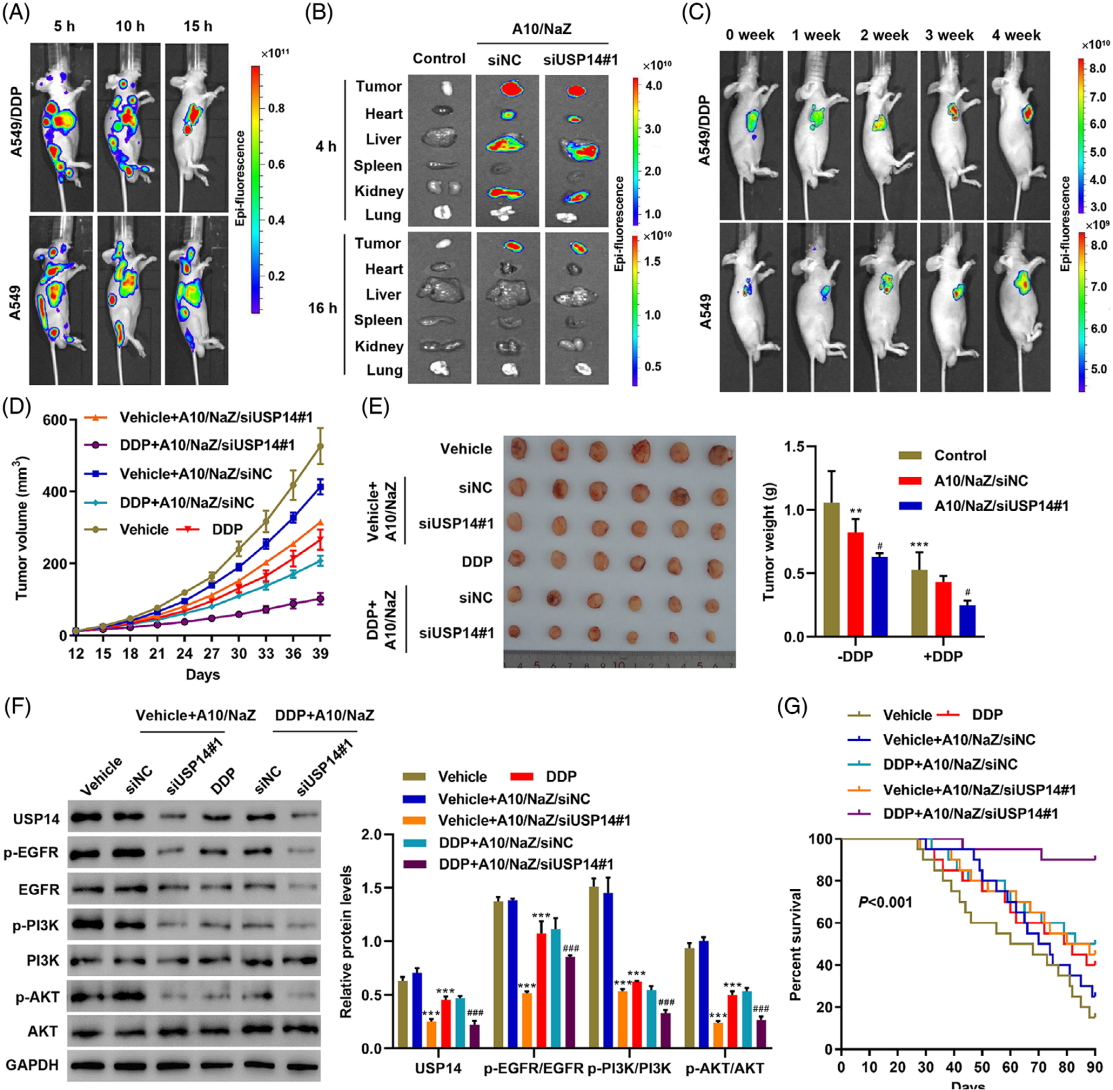
Antitumor efficacy of aptamer‐siRNA nano‐zinc carriers in a mouse lung cancer model bearing A549/DDP cells. A549/DDP or A549 cells were implanted subcutaneously in nude mice. After 10 days, mice received a single intravenous injection of 5 mg/kg/d A10/NaZ/siUSP14#1 followed by serial fluorescence imaging at the indicated time points. (A) Representative fluorescence imaging at different time points was visualized using Cy3‐labeled siUSP14#1. (B) Ex vivo fluorescence images of major tissues excised from mice bearing A549/DDP cells at different time points were visualized using Cy3‐labeled siUSP14#1. (C) Representative bioluminescence (A549/DDP or A549 cells) imaging. (D) Tumor volume, (E) weight, and (F) expression of USP14 and relative proteins in the EGFR pathways of mice bearing A549/DDP cells treated with 5 mg/kg/d A10/NaZ/siNC or A10/NaZ/siUSP14#1 and 50 mg/kg/week DDP. Data are presented as means ± SD (*n* = 6). (G) Survival rate of mice bearing A549/DDP cells after different treatments (*n* = 20). **P < 0.01, ***P < 0.001 compared with control. ^#^ *P* < 0.05, ^###^ *P* < 0.001 compared with A10/NaZ/siNC. DDP, Cisplatin.

To examine the antitumor efficacy of aptamer‐siRNA nano‐zinc carriers in lung cancer in vivo, A549/DDP or A549 tumor cells were implanted into nude mice. After 10 days, mice received a single intravenous injection of 5 mg/kg/d aptamer A10‐coupled nano‐zinc carriers loaded with siUSP14#1 (A10/NaZ/siUSP14#1). The distribution of Cy3‐labeled siUSP14#1 delivered by the A10/NaZ carriers was evaluated using biophotonic imaging technology. Whole body images showed a strong signal for A10/NaZ/siUSP14#1 in the heart, liver, and kidneys 5 and 10 h after injection (Figure 6A). A10/NaZ/siUSP14#1 accumulated in tumors, and the signal was stronger in mice bearing A549/DDP cells compared with mice bearing A549 cells (Figure 6A). The penetration depth of the optical source was limited in whole‐body imaging. Thus, mice bearing A549/DDP cells were sacrificed and the organs were excised for ex vivo imaging. Ex vivo fluorescent imaging showed a strong signal in tumors, heart, liver, and kidney 4 h after injection, while a fluorescent signal was detected in tumors but not other organs 16 h after injection (Figure 6B). Surviving A549/DDP and A549 cells were detected using bioluminescence at 0, 1, 2, 3, and 4 weeks after injection, verifying the in vivo targeting efficacy of A10/NaZ/siUSP14#1 (Figure 6C). These results demonstrate that aptamer‐siRNA nano‐zinc carriers inhibit tumor growth in vivo.

Different concentrations of the nano‐zinc carrier (0, 5, and 10 mg/kg/d) were intravenously injected into nude mice. Initially, mouse body weights decreased in the 10 mg/kg/d group (Figure S2A). The heart, liver, lungs, spleen, and kidneys were histologically analyzed 30 days after injection with the indicated concentrations of the nano‐zinc carrier. Some chronic toxicity was observed in the 10 mg/kg/d group, mainly in the liver and kidney (Figure S2B). Therefore, a concentration of 5 mg/kg/d was used in subsequent studies. The survival rate and tumor morphology and volume were compared. Treatment with A10/NaZ/siUSP14#1 combined with DDP induced the best therapeutic effects, including smaller tumor volume and weight (Figure 6D,E). Expression levels of USP14 and relative proteins in the EGFR pathways were also examined in the xenograft tumors of mice after different treatments (Figure 6F). The survival rate of mice after different treatments is shown in Figure 6G. The changes in survival may be related to the improved pro‐apoptotic effects of DDP after nano‐zinc carriers released siUSP14#1 into cells (Figure S3A,B). These results demonstrate that aptamer‐siRNA nano‐zinc carriers inhibit DDP resistance in vivo.

## 3 DISCUSSION

Treatment of lung cancer, the most common type of carcinoma, is difficult due to disease progression at the time of diagnosis, causing a high mortality rate.^1^, ^32^ Cisplatin (DDP), also known as cis‐diaminodichloplatin, is a powerful nonspecific drug blocking the cell cycle.^33^, ^34^ DDP can enter cells through the passive transport. After entering cells, DDP binds to DNA and forms DNA‐platinum adducts, which disrupt DNA synthesis and mitosis in cancer cells.^35^, ^36^ The main anticancer molecular mechanisms of DDP include: (1) induction of oxidative stress characterized by reactive oxygen species and lipid peroxidation; (2) induction of the p53 signaling pathway and cell cycle arrest; (3) down‐regulation of proto‐oncogene and anti‐apoptotic protein expression; and (4) activation of the exogenous apoptosis pathway mediated by death receptors and the endogenous apoptosis pathway mediated by mitochondrial injury.^37^, ^38^, ^39^

Previous studies demonstrated increased USP14 mRNA expression levels in lung adenocarcinoma and the association of higher USP14 levels with worse prognosis.^40^ Similar high USP14 expression levels were observed in epithelial ovarian cancer, multiple myeloma, intrahepatic cholangiocarcinoma, and other tumors.^40^, ^41^ USP14 inhibits apoptosis by inhibiting the bcl‐XL signaling pathway and promotes proliferation by stimulating the Wnt signaling pathway in multiple myeloma.^42^ In this study, USP14 was associated with the occurrence of lung cancer and DDP‐resistant lung cancer. In addition, increased USP14 diminished the sensitivity of A549 cells to DDP, and siUSP14 treatment renewed the sensitivity of DDP‐resistant A549 cells to DDP. EGFR is crucial for DDP resistance.^43^ In DDP‐resistant A549 cells, siUSP14 intervention gradually downregulated intracellular EGFR and p‐AKT levels. EGFR is over‐expressed or abnormally expressed in many solid tumors.^44^, ^45^ EGFR affects proliferation, angiogenesis, tumor invasion, metastasis, and apoptosis.^46^, ^47^ Two signal transduction pathways play important roles downstream of EGFR: the Ras/Raf/MEK/ERK‐MAPK pathway and the PI3K/AKT/mTOR pathway.^48^, ^49^ Our data showed that USP14 interacts with and induces DUB of EGFR and activates the AKT signaling pathway.

Intervention in USP14 expression significantly improved DDP resistance in A549 cells. Thus, accurately targeting siUSP14 to drug‐resistant lung cancer cells to reverse drug resistance is worthwhile. Adaptors are an excellent solution to this problem; adaptors are convenient, fast, economical, and easy to manufacture and modify. AMs can be obtained by in vitro screening; thus, they can target specific application environments or molecular characteristics. Cell‐SELEX targets whole cells. The cell surface displays a complex variety of proteins, and AMs for various targets can be eliminated using cell‐SELEX. Molecules on the cell surface exhibit natural conformations and patterns. One advantage of whole‐cell screening is that the conformation of the target molecule does not need to be known in advance, and the destruction of target substances during purification can be avoided. In addition, screening for unknown molecular AM targets can also be performed.^50^, ^51^, ^52^ This study focused on the differential screening of cell membranes in A549/DDP and A549 cells. Thus, AMs specifically recognizing A549/DDP cells were obtained.

Porous nano‐zinc hydroxide has the advantageous abilities to load multiple compounds (directed AM and therapeutic drugs, such as nucleic acids) and to be easily internalized.^53^ After entering cells, nano‐zinc hydroxide exerts toxic effects by inducing oxidative stress, Zn^2+^ release, mitochondrial dysfunction, endoplasmic reticulum stress, inflammatory response, and DNA damage.^54^ To take advantage of these properties, the selected aptamer A10 was coupled to the surface of nano‐zinc hydroxide to target the molecules, and siUSP14 was encapsulated. Thus, siUSP14 entry into cells is increased by the maximally efficient nanocarriers. After nanocarriers enter the targeted cells, siUSP14 is released due to pH changes inside the cells. We also verified the implementation of this system in vivo using a tumor‐bearing nude mouse model.

Our study inevitably has some limitations. Limitations include our small‐sample size and insufficient follow‐up data. In addition, more research is still needed to further clarify the potential mechanism by which USP14 regulates DDP resistance in both in vitro and in vivo models of NSCLC.

In conclusion, differential ligands of the two types of cells were isolated by Cell‐SELEX, and the ligands were used as guiding elements. The ligands were combined with nano‐zinc hydroxide carriers to guide AMs and siRNA to target DDP‐resistant cells. After internalization of the paired AMs and carriers, intracellular pH changes cause the active release of the siRNA (Figure S4). Moreover, cell and animal experiments support the great application potential of this method, especially for precise intracellular therapeutic strategies.

## 4 MATERIALS AND METHODS

### 4.1 Bioinformatics analysis

Gene expression information was gathered from TCGA database for lung cancer, which included 526 cases of tumor tissues and 59 cases of normal lung tissues. The GSEA algorithm was employed to identify pathways that were strongly supplemented in response to higher USP14 expression.

### 4.2 Clinical samples

Sixty samples with lung cancer for whom the first line drug therapy was DDP were selected from Fudan University Shanghai Cancer Center from July 2020 to October 2021. The samples were categorized into four groups based on the difference in tumor volume following treatment, as follows: (1) complete response group (CR) who had no tumor left, (2) partial response group (PR) who showed tumor shrinkage of > 50%, (3) stable disease group (SD) for whom the tumor shrinkage was < 50% or tumor enlargement was > 25%, and (4) progressive disease (PD) for whom tumor grew by > 25% or more. CR along with PR was identified as chemotherapy sensitive cases, whereas SD and PD were considered chemotherapy‐resistant cases. Following informed consent, tissues were collected and frozen in liquid nitrogen until use. All procedures were approved by the Institutional Ethical Committee at Fudan University Shanghai Cancer Center (approval number: 2004216‐19‐2005). All patients who donated tissues provided informed consent.

### 4.3 Immunohistochemistry

Primary lung cancer tissues were embedded in paraffin solution and sectioned for IHC analysis. The specimens were stained with an anti‐USP14 antibody (ab137433; Abcam) followed by HRP‐conjugated anti‐IgG antibody (Long Island Biotech, China; D‐3004), using the standard protocol. IHC analysis was conducted by two independent pathologists who were blinded to the clinical and pathological features of the cases.

### 4.4 Cell culture and transfection

The A549 (ATCC, Manassas, VA, USA; CRM‐CCL‐185), A549 cisplatin‐resistant (A549/DDP; National Infrastructure of Cell Line Resource, Beijing, China; 1101HUM‐PUMC000519), and human lung bronchial epithelial 16HBE cells (Shanghai Fuheng Biotechnology Co., Ltd. China; FH1013) were cultured in RPMI 1640 medium (Hyclone, Logan, UT, USA) with 10% fetal bovine serum and 1% penicillin/streptomycin (Life Technologies) in an atmosphere of 5% carbon dioxide and 95% air at 37°C.

Lung cancer cells were transfected with USP14 siRNA (siUSP14#1 and siUSP14#2) or nonspecific siRNA (siNC) (all from Shanghai GenePharma Co., Ltd, China) using DharmaFECT one siRNA infection reagent (Thermo Fisher Scientific). The siRNA sequences were: siUSP14#1: GACAGAAAGUUAUGGUGAAAG; siUSP14#2: AGUUCUUAAGGAUGUUAAAUU; siNC: GGACGAGCUGUACAAGUAA. For USP14 overexpression, the coding sequence was synthesized and cloned into pLVX‐Puro plasmids (TSPLA16346, Testobio Co., Ltd, Ningbo, China). Recombinant plasmids, along with psPAX2 (#12260) and pMD2.G (#12259) packaging plasmids (all from Addgene Headquarters, Watertown, MA, USA), were co‐transfected into 293T cells (ATCC; CRL‐3216) by using Lipofectamine 2000 (Thermo Fisher Scientific). At 48 h post‐transfection, recombined vectors were used for cell transduction. The empty vector and nonspecific siRNA acted as negative controls.

### 4.5 Cell counting kit‐8 assay

Cell viability was measured using the CCK‐8 (SAB Biotech., College Park, MD, USA) assay. Cells were cultured in 96‐well plates at 3 × 10^3^ cells/well for 12 h. Following treatment for 48 h, the CCK‐8 reagent was added to each well and incubated for 1 h. Absorption was measured at 450 nm.

### 4.6 Cell apoptosis assay

Cells were seeded into 6‐well plates (5 × 10^5^/well) and grown to 50% confluency. After treatment, cell apoptosis was analyzed by flow cytometry. Cells were incubated with 5 μL fluorescein isothiocyanate‐labeled recombinant annexin V (Annexin V‐FITC) for 15 min in the dark at 4°C and then incubated with 5 μL PI for 15 min. FACScan flow cytometry (Becton Dickinson, Franklin Lakes, NJ) with CellQuest software (Becton Dickinson) was performed to assess cell apoptosis.

### 4.7 Co‐immunoprecipitation and ubiquitination assay

Cells were lysed with radioimmunoprecipitation assay (RIPA) buffer and incubated with anti‐USP14 (ab70311; Abcam), anti‐EGFR (ab52894; Abcam), or normal IgG (sc‐2027; Santa Cruz Biotechnology, Inc.) antibodies and then incubated with protein A/G PLUS‐Agarose beads (sc‐2003; Santa Cruz Biotechnology, Inc.) for 2 h at 4°C. The immune complex was washed thrice with lysis buffer. Proteins were then detected with standard Western blot analysis using anti‐USP14 (ab175810; Abcam), anti‐EGFR (ab32077; Abcam), and anti‐ubiquitin (ab7780; Abcam) antibodies.

### 4.8 Quantitative real‐time polymerase chain reaction

Total RNA was isolated from lung cancer tissues and cell lines using Trizol reagent (Invitrogen, USA) following the manufacturer's guidelines and reverse‐transcribed with a RevertAid First Strand cDNA Synthesis Kit (Thermo Fisher, USA). Quantitative RT‐PCR was performed with SYBR green PCR Master Mix (Thermo Fisher) on an ABI7300 system. The forward and reverse primers used for qRT‐PCR were as follows: USP14 (5′‐AGGTCATTATGTATCATGGGTG‐3′ and 5′‐ATTTCAACTCTGCGAGGC‐3′); EGFR (5′‐GAAGAAGACATGGACGACG‐3′ and 5′‐TGTATTCAGGCACTGGGAG‐3′); GAPDH (5′‐GGATTGTCTGGCAGTAGCC‐3′ and 5′‐ATTGTGAAAGGCAGGGAG‐3′). The relative abundance of genes was quantified using the comparative 2^−ΔΔ^ *^Ct^* method with GAPDH as an internal control.

### 4.9 Western blot

Cells were lysed in RIPA buffer supplemented with fresh protease inhibitor cocktail (Sigma, St. Louis, MO, USA). Proteins were separated by sodium dodecyl sulphate‐polyacrylamide gel electrophoresis (SDS‐PAGE) and transferred onto nitrocellulose membranes (Millipore, Bedford, USA). Membranes were blocked with 5% skim milk and incubated with primary antibodies against USP14 (ab235960; Abcam), p‐EGFR (ab182618; Abcam), EGFR (#4267; CST), p‐PI3K (ab245781; Abcam), PI3K (ab191606; Abcam), AKT (#4685; CST), p‐AKT (#4060; CST), PCNA (ab29; Abcam), cleaved caspase‐3 (ab214430; Abcam), and GAPDH (#5174; CST) followed by an HRP‐conjugated secondary antibody (A0208; Beyotime, Shanghai, China). Antibody binding was detected with enhanced chemiluminescence (Bio‐Rad, Richmond, CA, USA).

### 4.10 Cell‐SELEX

A549/DDP and A549 cells were grown to the logarithmic growth stage. A549/DDP cells were adjusted to 1 × 10^7^ and washed three times with pre‐cooled sterile PBS. Sequences for the AM libraries and primers were as follows: Library (5′‐GAATTCCAGAGTGACGCAGCA‐(45N)‐TGGACACGGTGGCTTGAGCTC‐3′) (crossed sequences were EcoR I and Sac I restriction sites) and primers (P1: 5′‐GaattCCagAGTGACgCAGC‐3′; P2: 5′‐GAGCTCAAGCCACCGTGTCC‐3′). Library DNA (1000 pmol) was dissolved in binding buffer (Duchenne PBS; Sigma–Aldrich, St. Louis, MO), heated to 95°C, immediately placed on ice for 10 min, and incubated with A549/DDP cells at 37°C for 1 h. Cells were washed three times with Duchenne PBS. Then, the ssDNA bound to A549/DDP cells was eluted with elution buffer (0.1 M NaCl solution). NATCH‐5 column desalting (GE Healthcare) was used as a template, and primer P1 was used for asymmetric PCR amplification to obtain ssDNA in a new library round. The next round of screening was performed by binding to A549/DDP cells. In the fourth round, A549 cells were used as the reverse screening target. The ssDNA of unbound A549 cells was in the supernatant and was retained as the template, and the ssDNA was amplified by asymmetric PCR with primer P1 as the library for the next round of screening. After 12 rounds of forward screening and 6 rounds of directional screening, the resulting ssDNA was used as a template. Primers P1/P2 were added, and dsDNA was amplified by PCR. After digestion with restriction endonucleases, the dsDNA was inserted into the pUC19 plasmid and introduced into DH5ɑ cells. After overnight culture at 37°C, monoclonal colonies were selected. Extracted plasmids were identified by enzyme digestion. Biotin‐labeled ssDNA was amplified by asymmetric PCR using bio‐P1 primers and positive cloned plasmids as templates. The bio‐ssDNA from the same number of the clone sources was heated at 95°C for 5 min and immediately placed on ice for 10 min after denaturation. The bio‐ssDNA was mixed with A549/DDP and A549 cell lines respectively for 1 h, washed three times, and incubated with FITC coupled with avidin for 30 min. After washing three times, the AM binding was detected by flow cytometry. AMs with increased affinity and specificity were screened and their dissociation constants were determined. FITC‐labeled AM and unlabeled AM were synthesized according to the sequencing results and detected by flow cytometry and laser confocal microscope.

### 4.11 Preparation of nano‐zinc carrier and its coupling with Aptamers

To prepare the nano‐zinc carrier, 36 g sodium hydroxide, 70 mL deionized water, 563 μL EDA, and 34% hydrazine 263 μL were stirred vigorously for 15 min, and 4 mL of 0.1 M copper nitrate was added drop by drop to the solution with intense agitation. The liquid was transferred to a stainless steel autoclave lined with Teflon, and 2 g/L polyethylene pyrrolidone (1300 KD molecular weight) solution was gently added above the liquid level. After sealing, the solution was heated in an electric oven at 80°C for 3 h, and then cooled to room temperature. The solution was centrifuged and the resulting brown‐red copper nanoparticles were washed with deionized water and 0.01% hydrazine solution and vacuum dried overnight at 50°C. Nanocopper (6.35 mg), deionized water (6 mL), and ethanol (6 mL) were successively added to a 50 mL flat‐bottomed flask. After 15 min of ultrasound, 300 mg polyvinylpyrrolidone with a molecular weight of 24,000 was added to the solution. The solution was stirred vigorously for 15 min, then ZnCl_2_ (8 mg) in 0.6 mL aqueous solution was added. After stirring for 10 min, 4 mL (drops/2s) of 1 M Na_2_S_2_O_3_ aqueous solution was added to the mixture. Finally, after stirring for 10 min, the red mixture gradually turned transparent gray. The solid substance was collected by centrifugation and cleansed with acetone and methanol three times. The dry transparent gray solid substance (6 mg, nano‐zinc carrier) was dissolved in 15 mL toluene solution. After ultrasonication for 3 h, 15 μL of triethoxysilane was added and the mixture was stirred for 24 h. The solid matter was washed with ethanol, resulting in the aminoated nano‐zinc carrier. The aminoated nano‐zinc carrier was dispersed in 1 mL of Duchenne PBS. Sulfo‐ sulfosuccinimidyl 4‐(N‐maleimidomethyl)cyclohexane‐1‐carboxylate (1 mg) was dissolved in 50 μL DMSO, and then mixed with Duchenne PBS and shaken for 1 h. After centrifugation, the aminoated nano‐zinc carrier was dispersed in 0.5 mL Duchenne PBS, mixed with 100 μL AM (16 μmol/L), and shaken for 24 h.

### 4.12 Loading of Aptamers ‐coupled nano‐zinc carrier with USP14 siRNA

The AM‐coupled nano‐zinc carrier was mixed with 10 μM USP14 siRNA in Duchenne PBS at a weight ratio of 1/1 and incubated for 24 h.

To quantify siRNA loaded in nano‐zinc carriers, the nano‐zinc carriers loaded with USP14 siRNA at various weight ratios were centrifuged at 10,000 rpm for 15 min and separated on a 2% agarose gel in 0.5 × tris‐acetate‐EDTA (TAE) buffer at 80 V for 40 min. The gel was stained with ethidium bromide, and siRNA bands were detected at 302 nm using Azure C300 (Dublin, CA, USA) as previously described.^55^ The USP14 siRNA loading efficiency was calculated using the following formula: (band intensity of pellets of nano‐zinc carriers loaded with USP14 siRNA complex/band intensity of 10 μM of USP14 siRNA) × 100%.

To evaluate the protection of the USP14 siRNA against RNase A digestion, 5 pmol of free siRNA or nano‐zinc carriers loaded with USP14 siRNA complex was incubated with 5 mU RNase A for 2 h at 37°C. The samples were then separated by electrophoresis using a 2% agarose gel as described above.

### 4.13 Release of siRNA

Nano‐zinc carrier equivalent to 10 μM of USP14 siRNA was mixed in 100 μL of deionized water at pH 5.2, pH 6.2, or pH 7.4. The nano‐zinc carrier mixes were incubated at 37°C under continuous agitation and the suspension was sampled at selected time intervals, loaded on a 2% agarose gel, and run in 0.5 × TAE buffer at 80 V for 40 min. After staining with ethidium bromide, the siRNA bands were identified at 302 nm using Azure C300 (Dublin, CA, USA). The percent of USP14 siRNA release was calculated using the following formula: (band intensity of released USP14 siRNA/band intensity of 10 μM of USP14 siRNA) × 100%.

### 4.14 Targeting of the nano‐zinc carrier in vivo

Targeting of the nano‐zinc carrier to A549/DDP or A549 cells in vivo was detected after subcutaneous injection in a mouse xenograft tumor model. A549/DDP or A549 cells (1 × 10^6^ cells) in 50 μL of PBS were injected into the armpits of 4‐week‐old nude mice (BALB/c, nu/nu; SIPPR‐BK Laboratory Animal Co. Ltd, Shanghai, China) using a 25‐gauge needle. After allowing tumors to form for 10 days, 5 mg/kg/d of the nano‐zinc carrier‐AM‐USP14 siRNA complex was introduced via the tail vein for 4 weeks. Cy3‐labeled USP14 siRNA fluorescent images were obtained using the Caliper IVIS Lumina III (Perkin Elmer, USA) at 5, 10, and 15 h to verify biodistribution of the nano‐zinc carrier‐AM‐USP14 siRNA complex. Survival of the A549/DDP and A549 cells in vivo was measured using bioluminescence at 0, 1, 2, 3, and 4 weeks to verify the in vivo targeting efficacy of the nano‐zinc carrier. An intraperitoneal injection of DDP (50 mg/kg/week) was administered on day 12 post‐injection. Tumor volume was calculated every 3 days using the following equation: Volume = (length × width^2^)/2. After anesthetizing with 3% isoflurane, mice were sacrificed by cervical dislocation on day 39, and the liver, heart, lungs, kidneys, spleen, and xenograft tumors were collected, washed, fixed with 4% paraformaldehyde, and processed to produce paraffin‐embedded sections. To evaluate toxicity and apoptosis, sections were stained with hematoxylin and eosin or terminal‐deoxynucleoitidyl transferase‐mediated nick end labeling (TUNEL; Sigma, Shanghai, China). Another 120 mice were collected for survival analysis for 90 days. All animal procedures were approved by the Animal Care and Use Committee of Shanghai Rat@Mouse Biotech Co., Ltd., China (approval number: 20210318).

### 4.15 Statistical analysis

All experiments were performed three times and the quantitative data are presented as mean ± standard deviation. GraphPad Prism version 8.4.2 (GraphPad Software, San Diego, CA, USA) was used to compute statistics. Variables were compared using unpaired *t*‐tests or analysis of variance (ANOVA) followed by post hoc analyses using Tukey's post‐multiple test. The significance level for the study was fixed at *P* < 0.05.

## AUTHOR CONTRIBUTIONS

Xinmin Zhao and Xianghua Wu designed the study. Xianghua Wu and Jialei Wang performed the experiments. Xianghua Wu and Huijie Wang contributed to data analysis. Xinmin Zhao, Xianghua Wu, and Huijie Wang performed data interpretation. Xinmin Zhao, Songtao Lai, and Jialei Wang contributed to the critical revision of the manuscript. Xinmin Zhao wrote the manuscript. All authors approved the final version of the manuscript.

## CONFLICT OF INTEREST STATEMENT

The authors declare no conflicts of interest.

## ETHICS STATEMENT

All procedures were approved by the institutional Ethical Committee at Fudan University Shanghai Cancer Center (approval number: 2004216‐19‐2005). All patients who donated tissues provided the informed consent. This study was performed in accordance with the Declaration of Helsinki. All animal procedures were approved by the Animal Care and Use Committee of Shanghai Rat@Mouse Biotech Co., Ltd., China (approval number: 20210318).

## Supporting information

Supporting Information

## Data Availability

The data used to support the findings of this study are available from the corresponding author upon request.
