# Correction to: Targeted therapy for cisplatin‐resistant lung cancer via aptamer‐guided nano‐zinc carriers containing USP14 siRNA

**DOI:** 10.1002/mco2.385

**Published:** 2023-09-23

**Authors:** Xinmin Zhao, Xianghua Wu, Huijie Wang, Songtao Lai, Jialei Wang

**Affiliations:** ^1^ Department of Thoracic Medical Oncology Fudan University Shanghai Cancer Center Shanghai China; ^2^ Department of Oncology Shanghai Medical College Fudan University Shanghai China; ^3^ Department of Radiation Oncology Fudan University Shanghai Cancer Center Shanghai China; ^4^ Department of Radiotherapy Shanghai Key Laboratory of Radiation Oncology Shanghai China

Correction to: *MedComm*  https://doi.org/10.1002/mco2.237, published online 2023 Apr 6

In the process of checking the raw data,^1^ we noted that the images of A549 group in Figure 4F were misplaced by mistake. The corrected Figure 4F should be as shown below. The authors confirm that the conclusions of this paper are not affected and sincerely apologize for this error and any inconvenience that may have caused.

**FIGURE 4:**
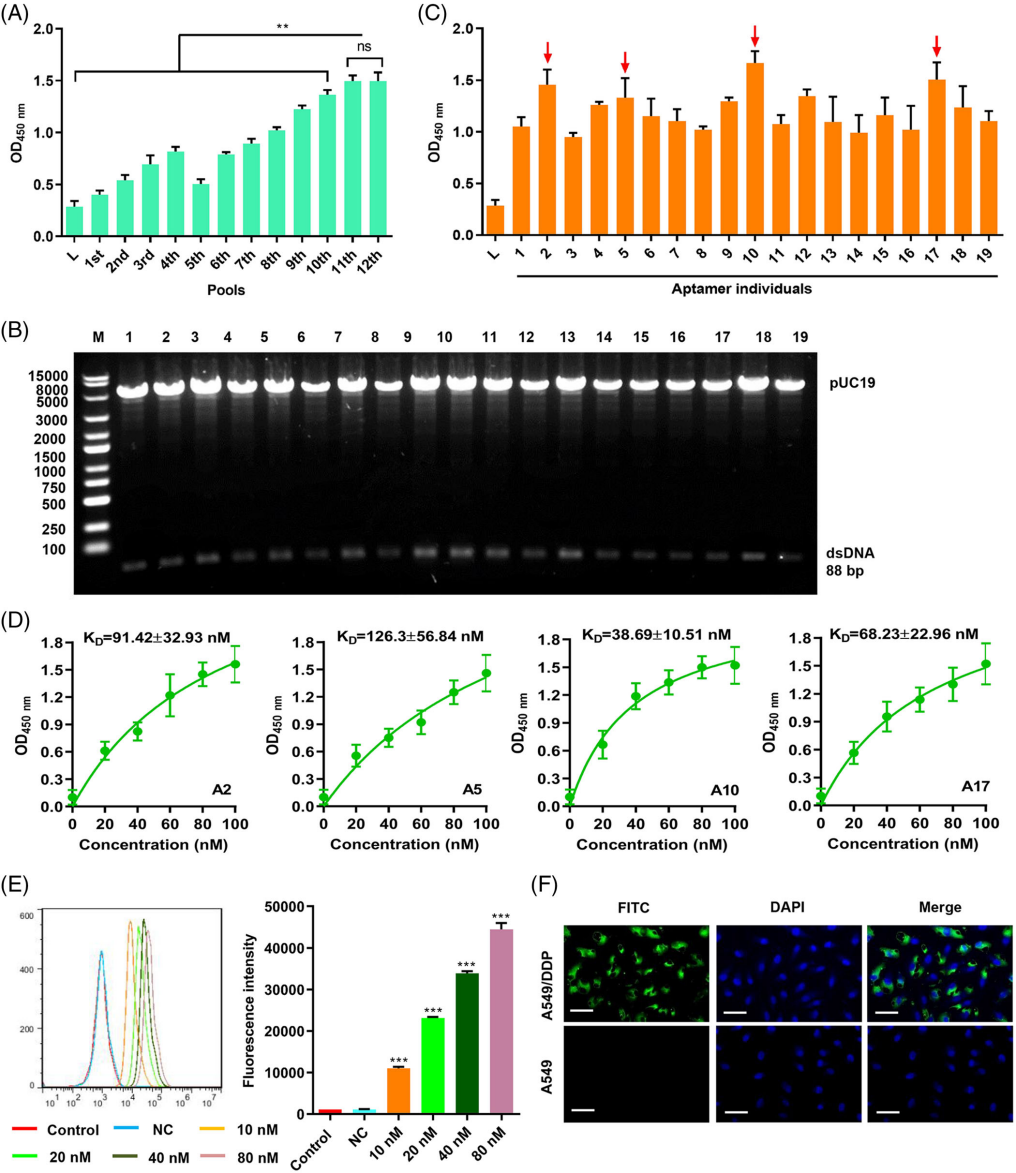
Cell‐SELEX for the identification of A549/DDP‐specific AM. (A) Binding capability of the enriched pools to A549/DDP cells determined by enzyme‐linked oligonucleotide assay. (B) Identification of dsDNA library amplified from the pool 11 library. (C) Binding of the AM candidates (A1–A19) to A549/DDP cells determined by enzyme‐linked oligonucleotide assay (red arrows indicated the AM candidates with higher binding forces). (D) Dissociation constant of aptamer candidates (A2, A5, A10, and A17) in A549/DDP cells was determined by enzyme‐linked oligonucleotide assay. (E) Binding of the different concentrations of FITC‐labeled aptamer 10 (A10) to A549/DDP cells detected by flow cytometry. (F) Subcellular localization of A10 in A549/DDP and A549 cells visualized using FITC‐labeled A10. Scale bar, 50 μm. Data are presented as means ± SD (*n* = 3). **p < 0.01, ***p < 0.001 in comparison with 11th, 12th, or NC.
