# Supplementary material for: Targeted therapy for cisplatin‐resistant lung cancer via aptamer‐guided nano‐zinc carriers containing USP14 siRNA
Source: MedComm (2020). 2023 Apr 6;4(2):e237. doi: 10.1002/mco2.237 (PMC10077057; doi:10.1002/mco2.237)
Supplement: Supplementary file 1 — Supporting Information [file MCO2-4-e237-s001.docx]

**Targeted therapy for cisplatin-resistant lung cancer via aptamer-guided nano-zinc carriers containing USP14 siRNA**

Xinmin Zhao^1,2^, Xianghua Wu^1,2^, Huijie Wang^1,2^, Songtao Lai^2,3,4,^*, Jialei Wang^1,2,^*

^1^Department of Thoracic Medical Oncology, Fudan University Shanghai Cancer Center, Shanghai, China

^2^Department of Oncology, Shanghai Medical College, Fudan University, Shanghai, China

^3^Department of Radiation Oncology, Fudan University Shanghai Cancer Center, Shanghai, China

^4^Shanghai Key Laboratory of Radiation Oncology, Shanghai, China

*Corresponding author

Jialei Wang, Department of Thoracic Medical Oncology, Fudan University Shanghai Cancer Center, No.270, Dong’An Road, Shanghai 200032, China. Tel: +8602164175590; E-mail: [m18017312369@163.com](mailto:m18017312369@163.com);

Songtao Lai, Department of Radiation Oncology, Fudan University Shanghai Cancer Center, No.270, Dong’An Road, Shanghai 200032, China. Tel: +08602165675209; E-mail: st.lai@shca.org.cn.

**Running title:** Precise treatment of aptamer-guided nano-zinc carriers in lung cancer

**
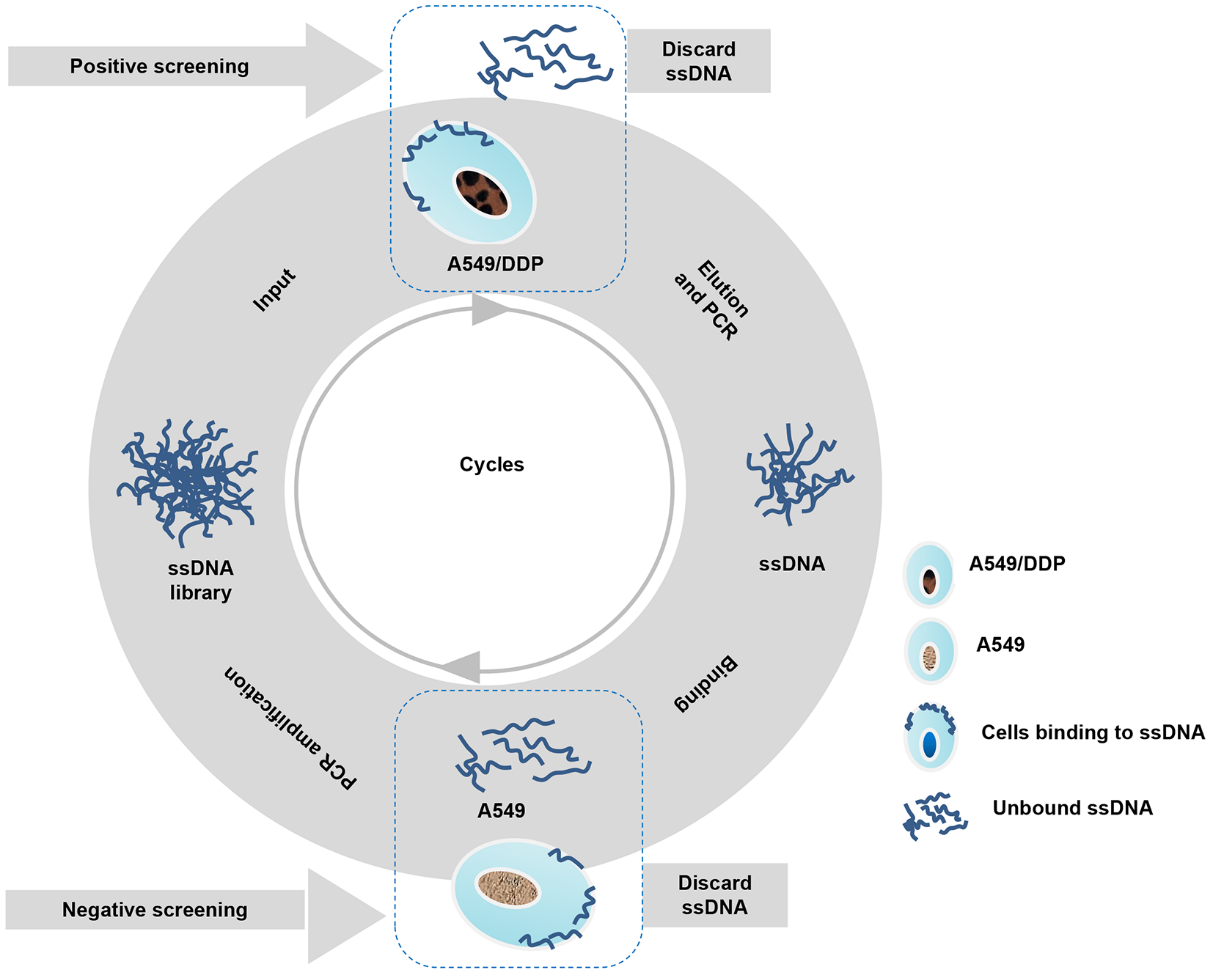
**

**Supplemental Figure S1. Illustration of the Cell-SELEX for the identification of A549/DDP-specific AM.**


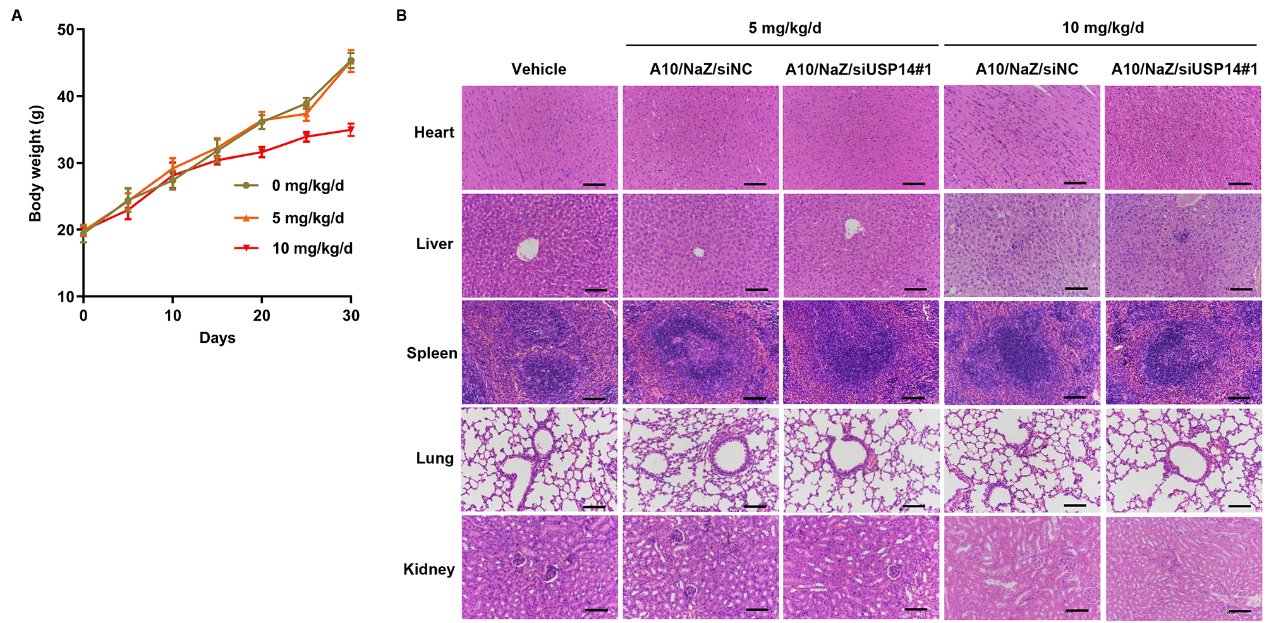


**Supplemental Figure S2. In vivo toxicity evaluation.** (A) Body weights of mice treated with 0, 5, 10 mg/kg/d A10/NaZ/siUSP14#1 for 30 days. (B) Images of H&E-stained sections obtained from the major organs (heart, liver, spleen, lung, and kidney) of mice 30 days post injection with 5 mg/kg/d or 10 mg/kg/d (A10/NaZ/siUSP14#1). Scale bar, 100 μm. Data are represented as mean ± SD (n=6).


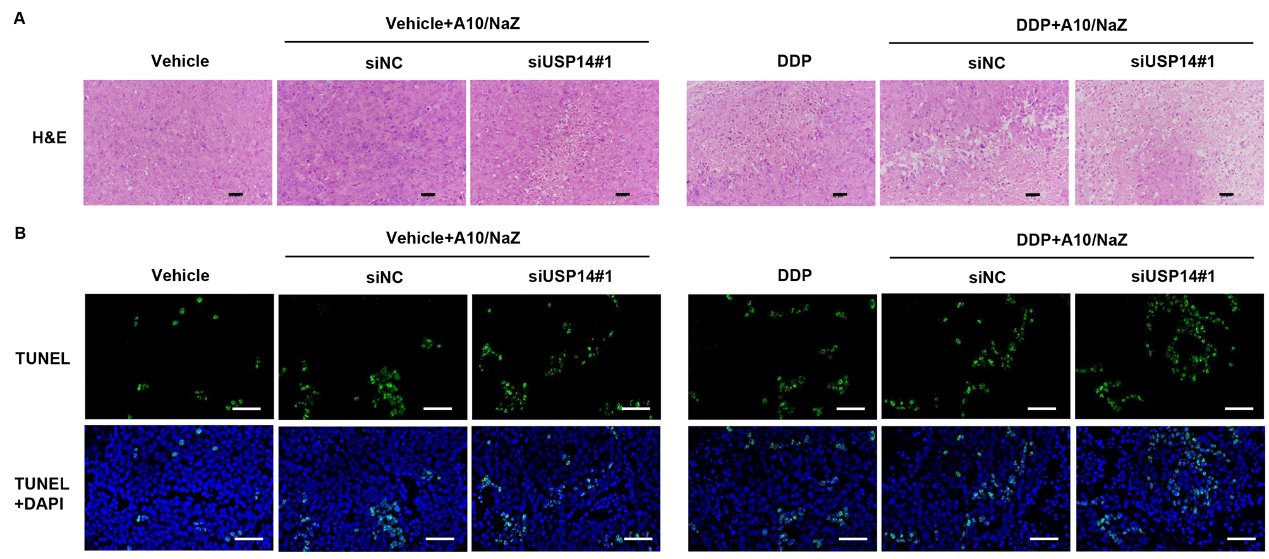


**Supplemental Figure S3. Pathology and apoptosis analysis in xenograft tumors of mice.** Nude mice bearing A549/DDP or A549 tumor were implanted subcutaneously. After 10 days, mice were received a single intravenous injection of 5 mg/kg/d A10/NaZ/siUSP14#1. (A) H&E and (B) TUNEL staining in xenograft tumors of mice treated with 5 mg/kg/d A10/NaZ/siNC or A10/NaZ/siUSP14#1 and 50 mg/kg/week DDP. Scale bar, 50 μm.


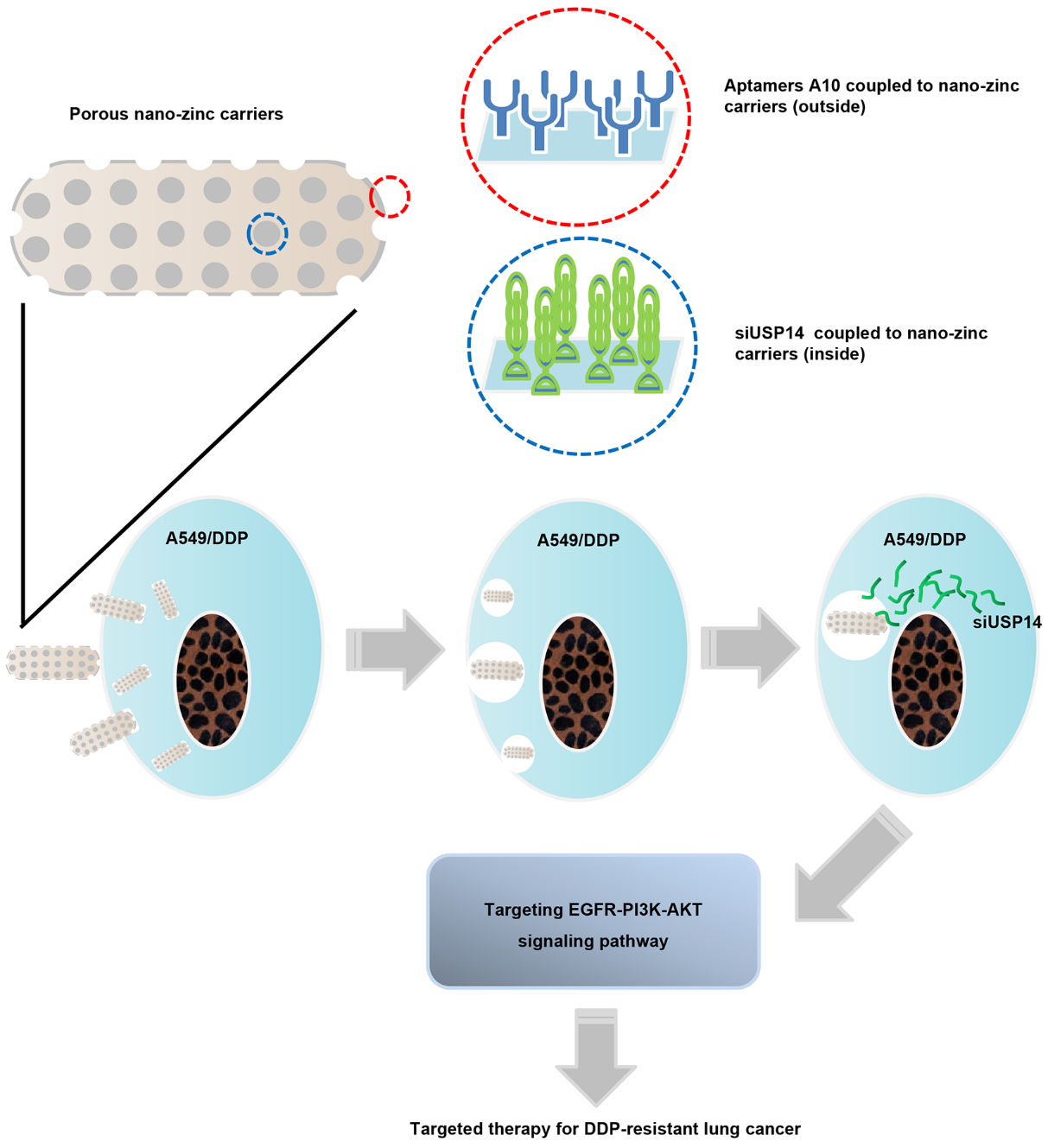


**Supplemental Figure S4. Illustration of the potential mechanism by which aptamer-guided Zinc nanocapsules containing siUSP14 ameliorate DDP resistance in lung cancer.** Aptamer-guided Zinc nanocapsules containing siUSP14 can specifically bind to the surface of A549/DDP cells, further release siUSP14 to suppress the protein expression of EGFR, followed by inactivation of PI3K/AKT signaling pathway, resulting in decreased DDP resistance in A549/DDP cells.
